# Evaluation of Crohn’s Disease Small-Bowel Mucosal Healing Using Capsule Endoscopy and Usefulness of Leucine-Rich α2-Glycoprotein

**DOI:** 10.3390/diagnostics13040626

**Published:** 2023-02-08

**Authors:** Hiroto Hiraga, Daisuke Chinda, Keisuke Hasui, Yasuhisa Murai, Takato Maeda, Naoki Higuchi, Kohei Ogasawara, Sae Kudo, Yohei Sawada, Tetsuya Tatsuta, Hidezumi Kikuchi, Mami Ebina, Noriko Hiraga, Tatsuya Mikami, Hirotake Sakuraba, Shinsaku Fukuda

**Affiliations:** 1 Department of Gastroenterology and Hematology, Hirosaki University Graduate School of Medicine, Hirosaki 036-8562, Japan; 2 Division of Endoscopy, Hirosaki University Hospital, Hirosaki 036-8563, Japan; 3 Center of Healthy Aging Innovation, Hirosaki University Graduate School of Medicine, Hirosaki 036-8562, Japan

**Keywords:** Crohn’s disease, small-bowel, mucosal healing, capsule endoscopy, leucine-rich α2-glycoprotein

## Abstract

Recently, the importance of achieving clinical and deep remissions with mucosal healing (MH) has been demonstrated as a therapeutic goal to avoid Crohn’s disease (CD) surgical operations. Although ileocolonoscopy (CS) is considered the gold standard, there are increasing reports on the benefits of capsule endoscopy (CE) and serum leucine-rich α2-glycoprotein (LRG) for evaluating small-bowel lesions in CD. We evaluated the data of 20 patients with CD who underwent CE in our department between July 2020 and June 2021 and whose serum LRG level was measured within 2 months. Concerning the mean LRG value, there was no significant difference between the CS-MH and CS-non-MH groups. Conversely, the mean LRG level was 10.0 μg/mL in seven patients in the CE-MH group and 15.2 μg/mL in 11 patients in the CE-non-MH group with a significant difference between the two groups (*p =* 0.0025). This study’s findings show that CE can sufficiently determine total MH in most cases, and LRG is useful for evaluating CD small-bowel MH because of its correlation with CE-MH. Furthermore, satisfying CS-MH criteria and a cut-off value of 13.4 μg/mL for LRG suggests its usefulness as a CD small-bowel MH marker, which could be incorporated into the treat-to-target strategy.

## 1. Introduction

Crohn’s disease (CD) is a chronic inflammatory disease of pan-enteric lesions, from the mouth to the anus, characterized by granulomatous inflammation of all layers and a discontinuous distribution. Patients with CD frequently require surgery because of stenosis, bleeding, abscess, and perianal disease [1]. Currently, the treatment and prognosis of CD are changing dramatically with the emergence of anti-TNF-α antibodies and other biological agents, such as anti-interleukin (IL)-12/23 antibodies and anti-α4β7 integrin antibodies.

Recently, the importance of achieving clinical and deep remissions with mucosal healing (MH) has been demonstrated as a therapeutic goal to avoid CD surgical operations [2]. Moreover, more attention has been paid to the treat-to-target (T2T) approach for CD [3]. T2T regards clinical remission as a short-term optimal goal and MH as a long-term goal and involves appropriate evaluation of disease activity using biomarkers and endoscopy. Although ileocolonoscopy (CS) is considered the gold standard for determining MH in CD, there are increasing reports on the usefulness of capsule endoscopy (CE) for evaluating small-bowel lesions in CD cases [4,5,6,7]. In patients with suspected CD, CE has shown good sensitivity (91–100%) and specificity (91–92%) when using CS as the standard [8,9]. In addition, CE has equivalent or higher diagnostic accuracy than other modalities in patients with established CD [7]. In patients with established CD, a meta-analysis reported significantly increased diagnostic accuracy with CE compared with small-bowel barium radiography (38%; 95% confidence interval (CI), 22–54%; *p* <0.00001) and computed tomography enterography (CTE) (32%; 95% CI, 16–47%; *p* <0.0001), but not CS (13%; 95% CI, −1–26%; *p* = 0.07) or magnetic resonance enterography (MRE) (−6%; 95% CI, −30–19%; *p* = 0.65) [7]. Furthermore, CE detected more lesions in the proximal small bowel than CTE or MRE [9,10]. A recent study reported that a high rate of mucosal lesions was identified during CE in patients with established CD who were in clinical remission [6]. Concerning the assessment of small-bowel MH, despite clinical remission, small-bowel MH was not detected on CE in any patient at Week 12; however, it was detected in 42% of patients at Week 52 [4,5]. The consensus group, all of whom were gastroenterologists practicing in Canada with expertise in CE, suggested that CE was a valid option in some patients, such as those with multiple resections or aggressive proximal or mid-small-bowel disease not within the reach of esophagogastroduodenoscopy or CS, where an assessment of the extent of ongoing disease activity may be warranted [11]. Moreover, CE provided additional information in 50–86% of patients; these additional findings influenced disease management and clinical outcomes in patients with established CD [12,13]. 

Noninvasive CD activity markers, such as C-reactive protein (CRP), erythrocyte sedimentation rate, blood albumin level (Alb), fecal calprotectin (FC), and CD Activity Index (CDAI), have been used to date; however, they may not accurately reflect the activity of CD small-bowel lesions [14,15,16]. FC correlates with endoscopic activity in inflammatory bowel disease (IBD) [17,18]. Although a cut-off value of 50 μg/g of FC can distinguish between IBD and non-IBD [19], there are no standard cut-off values of FC for MH in CD [20]. In addition, patients’ acceptance and resistance to sample transport have been reported [21,22] since the test is performed using feces. Leucine-rich α2-glycoprotein (LRG) is a 50 kDa glycoprotein that contains repetitive sequences with a leucine-rich motif [23,24], which is identified as a novel biomarker for rheumatoid arthritis and IBD using a proteomics approach [25]. LRG is expressed by hepatocytes, neutrophils, macrophages, and intestinal epithelial cells [26] and is induced by interleukin (IL)-22, tumor necrosis factor (TNF)-α, and IL-1β, independently from IL-6 [25]. Serum LRG has recently attracted attention as a more sensitive novel IBD marker as it correlates with the endoscopic activity of UC [27] and balloon-assisted endoscopic examination (BAE) of CD small-bowel lesions [28]. Although an increase in the serum LRG levels was observed in other diseases, such as rheumatoid arthritis, infection, and malignant diseases, the precise pathophysiology of LRG for IBD has not been clearly elucidated. Kawamoto et al. established that LRG is a sensitive biomarker for predicting intestinal ulcers in patients with CD and proposed a cut-off value of 13.4 μg/mL for LRG [28]. Recently, Omori et al. showed that LRG in quiescent CD might be useful in predicting the presence of small-bowel ulceration detected by CE [29] and proposed a cut-off value of 14 μg/mL for indicating the presence of small-bowel ulcerative lesions. However, few studies have been conducted concerning the comprehensive analysis of the correlation between the serum LRG levels and small-bowel MH using CE. 

We hypothesized that CE might be beneficial for CD small-bowel MH and LRG could be useful as a CD small-bowel MH marker. The present study aimed to investigate the usefulness of LRG as a CD small-bowel MH marker by analyzing the correlation between CD small-bowel MH evaluation using CE and serum LRG.

## 2. Materials and Methods

### 2.1. Study Design and Patients

**Figure 1:**
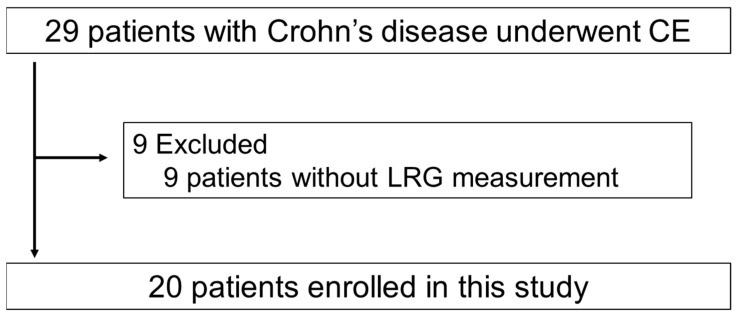
Flow chart of study patient selection.

The study included 29 consecutive patients with CD who underwent CE in our department between July 2020 and June 2021. Cases of incomplete CE, in which the cecum was not reached, and inaccessibility of videos due to mechanical errors were excluded from the study. We excluded nine patients without LRG measurements. Finally, 20 patients were included in the study (Figure 1). Table 1 and Table S1 show the patient characteristics. The serum LRG levels were measured in all 20 patients within 2 months and were analyzed by LSI Medience (Tokyo, Japan). CS was performed within 2 months in 12 of the 20 cases. Clinical disease activity was assessed using the CDAI, and clinical remission was defined by an index of <150 [30]. The Lewis Score (LS) [31] and Capsule Endoscopy Crohn’s Disease Activity Index (CECDAI) [32] were used as the CE activity scores. The LS [31] divides the small bowel into three segments by transit time and assigns scores to endoscopic findings (i.e., mucosal edema, ulcers, and strictures) and characteristics of CD. Scores <135, 135–790, and >790 points indicate normal or MH, mild inflammation, and moderate-to-severe inflammation, respectively. Another score, known as the CECDAI score, divides the small bowel into two segments and includes the degree and extent of mucosal inflammation and the presence of strictures [33]. Scores <3.5, 3.5–5.8, and >5.8 points indicate normal or MH, mild-to-moderate inflammation, and moderate-to-severe inflammation, respectively. MH in CE was defined as an LS of <135 and CECDAI score <3.5 points. We defined the group that achieved MH in LS and CECDAI as the “CE-MH group” and the group that did not achieve MH as the “CE-non-MH group.” Furthermore, we defined the group that achieved MH within the CS observation range (terminal ileum-rectum) as the “CS-MH group” and the group that did not achieve MH as the “CS-non-MH group.” This study was approved by the Ethics Committee of Hirosaki University Graduate School of Medicine (approval number: 2019-110-1). We obtained written informed consent from all patients (or guardians) before inclusion in this study.

### 2.2. Capsule Endoscopy Procedure

Small-bowel patency was confirmed using patency capsules (PC; Medtronic, Minneapolis, MN, USA) in all patients before the CE procedure. The PillCam^TM^SB3 (Medtronic) was used for all patients. The patient was instructed to ingest the PC at 23:00 (or before going to bed), 2 days before the CE examination. The patient was allowed to take 20 mL of 0.75% sodium picosulfate hydrate and 34 g of magnesium citrate at 21:00 on the day before the CE examination in preparation for the procedure and fasted for 12 h after. At 9:00 on the CE examination day, after confirming small-bowel patency (confirmation of PC excretion without collapse or PC existence in the colon within 30–33 h), the patient ingested the CE with water after drinking dimethicone water. The patient was released after identification of the intestinal villi using a real-time viewer. Drinking water was permitted for 2 h, and a meal was permitted for 4 h after CE ingestion. At 12 h after ingestion, the data recorder was removed, and CE excretion was confirmed visually by the patient. All images were analyzed using the RAPID 8 software (Given Imaging, Dublin, Ireland) by two board-certified physicians of the Japanese Association for Capsule Endoscopy. The LS was incorporated into the RAPID 8 software.

### 2.3. Statistical Analysis

The CRP levels, Alb levels, LRG levels, CDAI scores, LS, and CECDAI scores were compared between the CS-MH and CS-non-MH groups and between the CE-MH and CE-non-MH groups; a two-tailed *t*-test was used to compare these parameters in these groups. Spearman’s correlation coefficient was used to analyze the correlation between LRG and CE activity scores (LS and CECDAI) and each disease activity marker (CRP, Alb, CDAI). *p*-values <0.05 were considered statistically significant. All analyses were performed using GraphPad PRISM7 software (San Diego, CA, USA). 

## 3. Results

### 3.1. Patient Characteristics

The clinical and demographic characteristics of patients are shown in Table 1. The study included 20 patients (12 male and eight female patients) with a mean age of 33 (14–49) years. The disease locations were as follows: three had an ileal type, 15 had the ileocolonic type, and two had the colonic type. According to clinical activity assessed using CDAI, 16, 2, 1, and 1 patients were in remission, had mild diseases, had moderate disease, and had severe disease, respectively, with a mean CDAI score of 97.2 (20–327) points. The mean Alb, CRP, and LRG levels were 4.31 (3.6–5.1) g/dL, 0.155 (0.02–1.33) mg/dL, and 13.32 (8.5–22.2) μg/mL, respectively. CS was performed within 2 months in 12 of 20 cases, and CS-MH was achieved in five of 12 cases (41.7%). CE-MH was achieved in nine of 20 cases (45%), with mean LS and CECDAI scores of 642.8 (0–3789) and 6.3 (0–18), respectively. 

### 3.2. Connection between Crohn’s Disease Activity Markers and the Achieving Mucosal Healing in Ileocolonoscopy 

**Figure 2:**
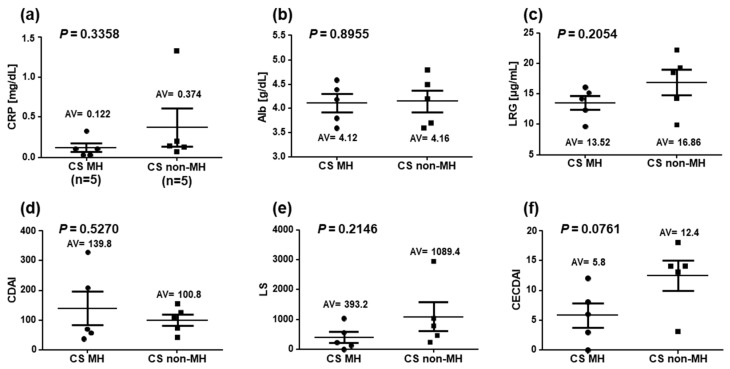
Connection between Crohn’s disease activity markers and mucosal healing in ileocolonoscopy. (**a**) CRP, (**b**) Alb, (**c**) LRG, (**d**) CDAI, (**e**) LS, and (**f**) CECDAI were determined in the CS-MH (*n* = 5) and CS-non-MH groups (*n* = 5); P-value is the result of comparing two groups by the two-tailed *t*-test. Abbreviations: CDAI: Crohn’s Disease Activity Index, Alb: blood albumin level, CRP: C-reactive protein, LRG: leucine-rich alpha-2 glycoprotein, LS: Lewis Score, CECDAI: Capsule Endoscopy Crohn’s Disease Activity Index, CS: ileocolonoscopy, MH: mucosal healing, CE: capsule endoscopy.

Three to five patients (60%) in the CS-MH group had active lesions in the small intestine, and none of the five patients (0%) in the CS-non-MH group (two cases of colonic type were excluded) achieved CE-MH. The mean LRG was 13.52 (9.7–16.1) μg/mL in the CS-MH group and 16.86 (9.9–22.2) μg/mL in the CS-non-MH group, with no significant difference between the two groups (*p =* 0.254). Other disease activity markers (CRP, Alb, CDAI, LS, and CECDAI) also showed no significant difference between the two groups (Figure 2).

### 3.3. Connection between Crohn’s Disease Activity Markers and the Achievement of Mucosal Healing in Capsule Endoscopy 

**Figure 3:**
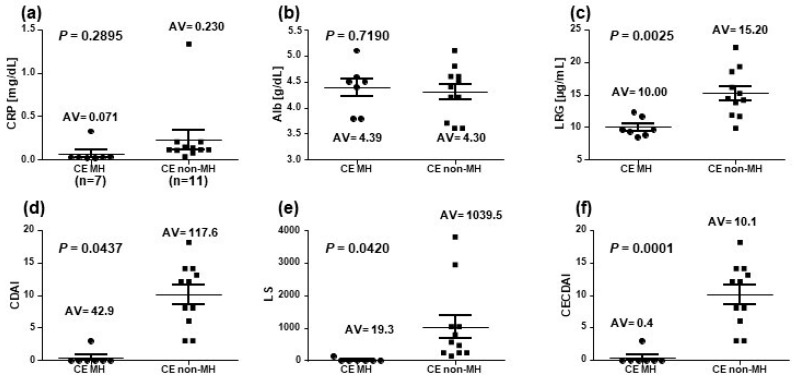
Connection between Crohn’s disease activity markers and mucosal healing in capsule endoscopy. (**a**) CRP, (**b**) Alb, (**c**) LRG, (**d**) CDAI, (**e**) LS, and (**f**) CECDAI were determined in the CE-MH group (*n* = 7) and CE-non-MH group (*n* = 11); P-value is the result of comparing two groups by the two-tailed *t*-test.

In the CE-MH group (two colonic types were excluded), CS was performed in two of the seven patients; all patients achieved CS-MH. Among the 11 patients in the CE-non-MH group, five of the eight patients (62.5%) who underwent CS were CS-non-MH. The mean LRG level was 10.0 μg/mL in seven patients of the CE-MH group and 15.2 μg/mL in 11 patients of the CE-non-MH group with a significant difference between the two groups (*p =* 0.0025). Furthermore, the mean CECDAI was 0.43 (0–3) in the CE-MH group and 10.09 (3–18) in the CE-non-MH group with a significant difference between the two groups (*p =* 0.0001). In addition, there was a significant difference in CDAI and LS between the two groups (*p =* 0.0437 and *p =* 0.042, respectively). Conversely, there was no significant difference in the CRP (*p =* 0.2895) and Alb (*p =* 0.7190) levels between the two groups (Figure 3). 

### 3.4. Correlation of Leucine-Rich α2-Glycoprotein with Crohn’s Disease Activity Markers 

**Figure 4:**
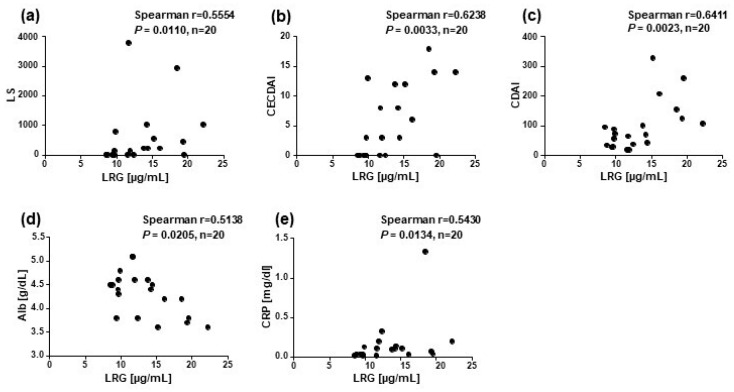
Correlations between the LRG levels and Crohn’s disease activity markers. The correlations between the LRG level and (**a**) LS, (**b**) CECDAI score, (**c**) CDAI score, (**d**) Alb level, and (**e**) CRP level were examined (*n* = 20); P-value is the result by Spearman’s correlation coefficient.

Figure 4 shows a significant correlation coefficient of r = 0.56 between the LRG levels and LS and r = 0.62 between the LRG levels and CECDAI values. LRG showed significant correlations with all disease activity markers: *r* = 0.5430 between the LRG and CRP levels, *r* = 0.5138 between the LRG and Alb levels, and *r* = 0.6411 between the LRG levels and CDAI score.

**Figure 5:**
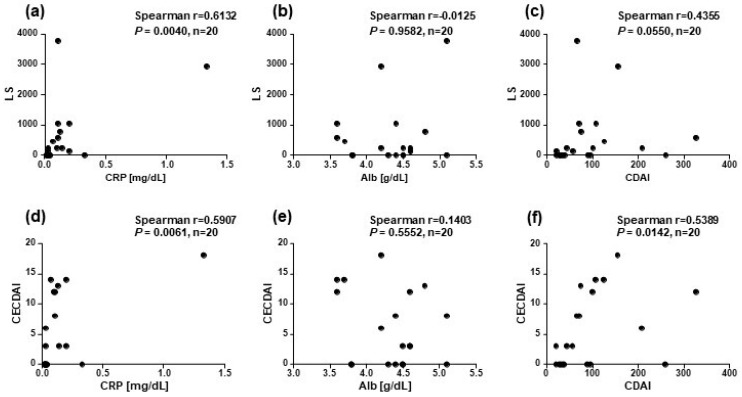
Correlation of CE scores with Crohn’s disease activity markers. The correlations between the LS and (**a**) CRP level, (**b**) Alb level, and (**c**) CDAI score was examined (*n* = 20). A correlation between the CECDAI score and (**d**) CRP level, (**e**) Alb level, and (**f**) CDAI score was examined (*n* = 20); P-value is the result by Spearman’s correlation coefficient.

As shown in Figure 5, a significant correlation was observed between the CRP levels and LS (*r* = 0.6132) but not between the Alb levels and LS (r = 0.01253) and between the CDAI score and LS (r = 0.4355). Concerning CECDAI, a significant correlation was observed between the CRP levels and CECDAI score (r = 0.5907) and between the CDAI and CECDAI (r = 0.5389) scores but not between the Alb levels and CECDAI score (r = 0.1403).

### 3.5. The Original Pictures of CE Findings 

**Figure 6:**
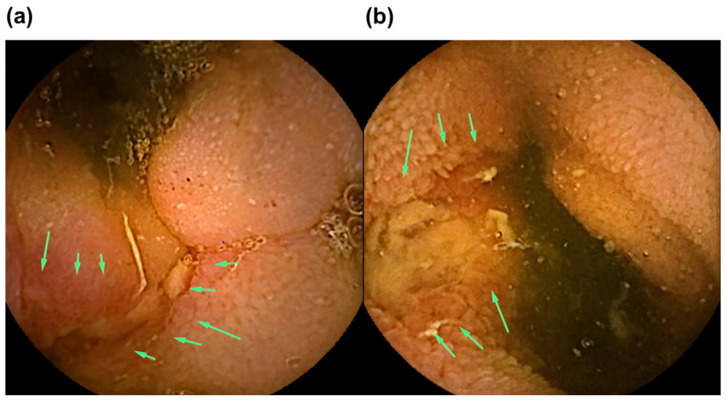
The original pictures of CE findings. (**a**,**b**) Distal ileum: linear ulcers (green arrows).

Figure 6 shows original pictures of CE findings in a 42-year-old male patient (Table S1). We present this case as an example that the LRG level is useful as a CD small-bowel MH marker to decide concerning the next treatment. In May 2021, the patient underwent emergency surgery for acute abdomen. As the intraoperative findings showed stricture with a longitudinal ulcer and perforation in the ileum, partial ileal resection was performed. CD was suspected on the pathological findings. However, findings of upper and lower gastrointestinal endoscopy were normal. He had no fever, abdominal pain, or anal lesions. He had 1–2 loose stools per day and remission in CD severity (CDAI score, 70 points). Laboratory tests indicated the following: white blood cell count, 5320/μL; CRP levels, 0.11 mg/dL; and LRG levels, 14.2 μg/mL. CE revealed active small-bowel lesions, such as notch-like depressions and longitudinal ulcer scars in the jejunum and aphthous and linear ulcers in the distal ileum (LS, 1036 points; CECDAI score, 8 (moderate–severe)).

## 4. Discussion

MH has become widely accepted as a therapeutic target for CD, as it is associated with reducing hospitalization and surgery. The findings of this study revealed that assessment using the existing disease activity markers and CS alone is insufficient for small-bowel MH determination. CE is useful for total MH determination that satisfies CS-MH and CE-MH as well as CD small-bowel MH determination. The LRG levels showed a significant correlation with disease activity markers, including the CE activity score. Moreover, satisfying CS-MH and a cut-off value of 13.4 μg/mL for LRG proposed by Kawamoto et al. was useful as an indicator of CD small-bowel MH or total MH [28].

Previous reports have shown that the CDAI score and CRP levels do not reflect CD small-bowel lesion activity [6,15]. Although the level of CRP, one of the major acute phase proteins synthesized in the liver, is induced by IL-6 [34], it may not always reflect disease activity in inflammatory diseases, such as systemic lupus erythematosus and ulcerative colitis (UC), which are primarily regulated by cytokines other than IL-6 [35]. In contrast, CS is still considered the gold standard for determining MH in CD. However, the indication of CS should be carefully considered because of its invasiveness and the possibility of accidental complications, which might result in disease aggravation [36]. Samuel et al. reported that CS does not provide sufficient information on the small bowel because it is almost impossible to observe the proximal side of the terminal ileum [14]. Concerning the activity scores using CS, the CD endoscopy index of severity [37] and the simple endoscopic score for CD (SES-CD) are generally used [38]; however, the risk of underestimating lesions in the proximal small intestine has been highlighted [14]. BAE and CE are also used to evaluate lesions in the proximal small bowel. Studies using BAE have reported that the presence of ≥0.5 cm ulcerative lesions is an independent risk factor for relapse and surgery [15], thus making it useful for small-bowel MH determination but unsuitable for frequent monitoring because of the increased invasiveness. In contrast, CE can be used to evaluate the entire small bowel with relatively minimal invasion in cases with confirmed small-bowel patency. A meta-analysis showed that CE has equivalent or higher diagnostic accuracy than other modalities in patients with suspected CD [7]. In this meta-analysis of 19 trials, there was a significant increment of diagnostic accuracy with CE, compared with CS (22%; 95% CI, 5–39%; *p* < 0.00001), radiography (32%; 95% CI, 16–48%; *p* < 0.00001), and CTE (47%; 95% CI, 31–63%; *p* = 0.009) but not MRE (10%; 95% CI, −14–34%; *p* = 0.43) [7]. More recently, two quantitative CE activity scores, LS and CECDAI, are available for monitoring CD. Although LS is derived from the most severe segment of the small bowel, CECDAI is a cumulative score that represents the summation of segmental scores for the proximal and distal small bowel. LS ≥ 350 points has been reported as a predictor of relapse within 24 weeks [39]; however, it has not been established as a method for evaluating small-bowel MH. In the present study, all patients in the CE-MH group satisfied CS-MH. In contrast, patients in the CS-non-MH group did not satisfy CE-MH, indicating that CE can detect CD small-bowel MH and total MH in most cases.

LRG reflects IBD activity more acutely than previous serum markers [26]. Several studies have evaluated LRG in patients with UC. Serada et al. reported an increased LRG level in patients with UC; the values correlated with clinical activity better than CRP [26]. Shinzaki et al. demonstrated that the serum LRG levels significantly increased and correlated with clinical and endoscopic activities in patients with UC [27]. Conversely, there are still few reports on the usefulness of LRG in detecting MH in patients with CD [40]. Yasutomi et al. showed that the correlations with the endoscopic activity and predictability of MH of LRG were similar to those of CRP and comparable to those of FC. In addition, it should be noted that the LRG levels discriminated patients with MH from those with endoscopic activity among patients with CD with normal CRP levels. However, the cut-off value for LRG as a CD small-bowel MH marker has not been established. LRG levels ≥16 μg/mL can reportedly differentiate between active (CDAI ≥ 150 points and SES-CD ≥4 points) or inactive CD (according to the manufacturer’s instructions; Sekisui Medical Co., Tokyo, Japan). Kawamoto et al. reported a cut-off value of 13.4 μg/mL for LRG as a predictor of ≥0.5-cm small-bowel ulcerative lesions using BAE [28]. Yasutomi et al. reported a cut-off value of 13 μg/mL as a predictor of SES-CD of 0 [40]. A study using CE proposed a cut-off value of 14 μg/mL as a predictor of LS < 350 points with 80% specificity and a negative predictive value of 100% [29]. Compared with the results obtained in other studies conducted on a larger number of patients [26,27,40], this study shows the same results that LRG could discriminate patients with MH from those with endoscopic activity among patients with IBD with normal CRP levels. In this study, the LRG levels showed a significant correlation with disease activity markers, including CE activity scores. Furthermore, satisfying CS-MH and a cut-off value of 13.4 μg/mL for LRG suggested its usefulness as an indicator of CD small-bowel MH and total MH. These results show that LRG is useful as a CD small-bowel MH marker that could be incorporated into T2T. Therefore, efficiently using LRG could reduce the invasion of patients with CD (a burden for the endoscopist) and the country’s medical expenses. 

This study has some limitations. First, this is a single-center study with a small sample size because the CE system is expensive, and our hospital is the only institution with a CE system in our medical area. In the future, multicenter studies are required. Although there was no recommended regimen for CE preparation, we performed CE according to the standard bowel cleansing preparation. Therefore, similar results would be obtained in multicenter studies. Second, this is a single-arm study without including a healthy control group; it was impossible to perform the exact measurement on healthy individuals. Third, the FC level was not measured in this study because the health insurance system in Japan did not cover its measurement. A further study of the relationship between the LRG and FC levels should be conducted, although a comparison of the accuracy of LRG and FC has been reported [40]. Fourth, the information concerning the lesions found in CE and its location was not examined in this study. A further study of the severity and location found in CE is required, as we know that patients with proximal small-bowel lesions have a worse prognosis.

## 5. Conclusions

The findings of our study showed that CS alone is insufficient to determine small-bowel MH, while CE can sufficiently determine total MH in most cases. CE is beneficial for CD small-bowel MH evaluation, and satisfying CS-MH and a cut-off value of 13.4 μg/mL for LRG suggests its usefulness as a CD small-bowel MH marker, which could be incorporated into the T2T approach.

## Figures and Tables

**Table 1:** Patient Characteristics.

Characteristics	
Sex (male: female)	12: 8
Age (years)	33 (14–49)
Disease location Ileal type Ileocolonic type Colonic type	3 (15) 15 (75) 2 (10)
CDAI Remission Mild Moderate Severe	97.2 (20–327) 16 (80) 2 (10) 1 (5) 1 (5)
Alb (g/dL)	4.31 (3.6–5.1)
CRP (mg/dL)	0.155 (0.02–1.33)
LRG (μg/mL)	13.32 (8.5–22.2)
CE activity scores LS CECDAI	571.8 (0–3789) 5.7 (0–18)
Achievement of MH CS-MH CE-MH	5/12 (41.7) 9/20 (45)

Data are presented as numbers (percentages) or medians (ranges). CDAI: Crohn’s Disease Activity Index, Alb: blood albumin level, CRP: C-reactive protein, LRG: leucine-rich alpha-2 glycoprotein, LS: Lewis Score, CECDAI: Capsule Endoscopy Crohn’s Disease Activity Index, CS: ileocolonoscopy, MH: mucosal healing, CE: capsule endoscopy. “CS-MH” means the group that achieved MH within the CS observation range (terminal ileum-rectum). “CE-MH” means the group that achieved MH in both LS < 135 and CECDAI < 3.5.

## Data Availability

The data presented in this study are available on request from the corresponding author. The data are not publicly available due to privacy and ethical restrictions.

## Supplementary Materials

The following supporting information can be downloaded at: https://www.mdpi.com/article/10.3390/diagnostics13040626/s1, Table S1: Patient Characteristics

## References

[1] D’Incà R., Caccaro R. (2014). Measuring disease activity in Crohn’s disease: What is currently available to the clinician. Clin. Exp. Gastroenterol..

[2] Lichtenstein G.R., Hanauer S.B., Sandborn W.J., Practice Parameters Committee of American College of Gastroenterology (2009). Management of Crohn’s disease in adults. Am. J. Gastroenterol..

[3] Turner D., Ricciuto A., Lewis A., D’Amico F., Dhaliwal J., Griffiths A.M., Bettenworth D., Sandborn W.J., Sands B.E., Reinisch W. (2021). STRIDE-II: An Update on the Selecting Therapeutic Targets in inflammatory bowel Disease (STRIDE) Initiative of the International Organization for the Study of IBD (IOIBD): Determining therapeutic goals for treat-to-target strategies in IBD. Gastroenterology.

[4] Hall B., Holleran G., Chin J.L., Smith S., Ryan B., Mahmud N., McNamara D. (2014). A prospective 52 week mucosal healing assessment of small bowel Crohn’s disease as detected by capsule endoscopy. J. Crohns Colitis..

[5] Hall B.J., Holleran G.E., Smith S.M., Mahmud N., McNamara D.A. (2014). A prospective 12-week mucosal healing assessment of small bowel Crohn’s disease as detected by capsule endoscopy. Eur. J. Gastroenterol. Hepatol..

[6] Kopylov U., Yablecovitch D., Lahat A., Neuman S., Levhar N., Greener T., Klang E., Rozendorn N., Amitai M.M., Ben-Horin S. (2015). Detection of small bowel mucosal healing and deep remission in patients with known small bowel Crohn’s disease using biomarkers, capsule endoscopy, and imaging. Am. J. Gastroenterol..

[7] Dionisio P.M., Gurudu S.R., Leighton J.A., Leontiadis G.I., Fleischer D.E., Hara A.K., Heigh R.I., Shiff A.D., Sharma V.K. (2010). Capsule endoscopy has a significantly higher diagnostic yield in patients with suspected and established small-bowel Crohn’s disease: A meta-analysis. Am. J. Gastroenterol..

[8] Casciani E., Masselli G., Di Nardo G., Polettini E., Bertini L., Oliva S., Floriani I., Cucchiara S., Gualdi G. (2011). MR enterography versus capsule endoscopy in paediatric patients with suspected Crohn’s disease. Eur. Radiol..

[9] Jensen M.D., Nathan T., Rafaelsen S.R., Kjeldsen J. (2011). Diagnostic accuracy of capsule endoscopy for small bowel Crohn’s disease is superior to that of MR enterography or CT enterography. Clin. Gastroenterol. Hepatol..

[10] Voderholzer W.A., Beinhoelzl J., Rogalla P., Murrer S., Schachschal G., Lochs H., Ortner M.A. (2005). Small bowel involvement in Crohn’s disease: A prospective comparison of wireless capsule endoscopy and computed tomography enteroclysis. Gut.

[11] Enns R.A., Hookey L., Armstrong D., Bernstein C.N., Heitman S.J., Teshima C., Leontiadis G.I., Tse F., Sadowski D. (2017). Clinical practice guidelines for the use of video capsule endoscopy. Gastroenterology.

[12] Gralnek I.M., Cohen S.A., Ephrath H., Napier A., Gobin T., Sherrod O., Lewis J. (2012). Small bowel capsule endoscopy impacts diagnosis and management of pediatric inflammatory bowel disease: A prospective study. Dig. Dis. Sci..

[13] Lorenzo-Zúñiga V., de Vega V.M., Domènech E., Cabré E., Mañosa M., Boix J. (2010). Impact of capsule endoscopy findings in the management of Crohn’s disease. Dig. Dis. Sci..

[14] Samuel S., Bruining D.H., Loftus E.V., Becker B., Fletcher J.G., Mandrekar J.N., Zinsmeister A.R., Sandborn W.J. (2012). Endoscopic skipping of the distal terminal ileum in Crohn’s disease can lead to negative results from ileocolonoscopy. Clin. Gastroenterol. Hepatol..

[15] Takenaka K., Ohtsuka K., Kitazume Y., Matsuoka K., Nagahori M., Fujii T., Saito E., Kimura M., Fujioka T., Watanabe M. (2018). Utility of magnetic resonance enterography for small bowel endoscopic healing in patients with Crohn’s disease. Am. J. Gastroenterol..

[16] Takenaka K., Ohtsuka K., Kitazume Y., Nagahori M., Fujii T., Saito E., Fujioka T., Matsuoka K., Naganuma M., Watanabe M. (2015). Correlation of the endoscopic and magnetic resonance scoring systems in the deep small intestine in Crohn’s disease. Inflamm Bowel. Dis..

[17] D’Haens G., Ferrante M., Vermeire S., Baert F., Noman M., Moortgat L., Geens P., Iwens D., Aerden I., Van Assche G. (2012). Fecal calprotectin is a surrogate marker for endoscopic lesions in inflammatory bowel disease. Inflamm. Bowel. Dis..

[18] Schoepfer A.M., Beglinger C., Straumann A., Safroneeva E., Romero Y., Armstrong D., Schmidt C., Trummler M., Pittet V., Vavricka S.R. (2013). Fecal calprotectin more accurately reflects endoscopic activity of ulcerative colitis than the Lichtiger Index, C-reactive protein, platelets, hemoglobin, and blood leukocytes. Inflamm. Bowel. Dis..

[19] Waugh N., Cummins E., Royle P., Kandala N.B., Shyangdan D., Arasaradnam R., Clar C., Johnston R. (2013). Faecal calprotectin testing for differentiating amongst inflammatory and non-inflammatory bowel diseases: Systematic review and economic evaluation. Health. Technol. Assess..

[20] Krzystek-Korpacka M., Kempiński R., Bromke M., Neubauer K. (2020). Biochemical biomarkers of mucosal healing for inflammatory bowel disease in adults. Diagnostics.

[21] Maréchal C., Aimone-Gastin I., Baumann C., Dirrenberger B., Guéant J.L., Peyrin-Biroulet L. (2017). Compliance with the faecal calprotectin test in patients with inflammatory bowel disease. United Eur. Gastroenterol. J..

[22] Zittan E., Gralnek I.M., Berns M.S. (2020). The new proactive approach and precision medicine in Crohn’s disease. Biomedicines.

[23] Haupt H., Baudner S. (1977). [Isolation and characterization of an unknown, leucine-rich 3.1-S-alpha2-glycoprotein from human serum (author’s transl)]. Hoppe. Seylers. Z. Physiol. Chem..

[24] Takahashi N., Takahashi Y., Putnam F.W. (1985). Periodicity of leucine and tandem repetition of a 24-amino acid segment in the primary structure of leucine-rich alpha 2-glycoprotein of human serum. Proc. Natl. Acad. Sci. USA.

[25] Serada S., Fujimoto M., Ogata A., Terabe F., Hirano T., Iijima H., Shinzaki S., Nishikawa T., Ohkawara T., Iwahori K. (2010). iTRAQ-based proteomic identification of leucine-rich alpha-2 glycoprotein as a novel inflammatory biomarker in autoimmune diseases. Ann. Rheum. Dis..

[26] Serada S., Fujimoto M., Terabe F., Iijima H., Shinzaki S., Matsuzaki S., Ohkawara T., Nezu R., Nakajima S., Kobayashi T. (2012). Serum leucine-rich alpha-2 glycoprotein is a disease activity biomarker in ulcerative colitis. Inflamm. Bowel. Dis..

[27] Shinzaki S., Matsuoka K., Iijima H., Mizuno S., Serada S., Fujimoto M., Arai N., Koyama N., Morii E., Watanabe M. (2017). Leucine-rich Alpha-2 glycoprotein is a serum biomarker of mucosal healing in ulcerative colitis. J. Crohns. Colitis.

[28] Kawamoto A., Takenaka K., Hibiya S., Ohtsuka K., Okamoto R., Watanabe M. (2022). Serum leucine-rich alpha2 glycoprotein: A novel biomarker for small bowel mucosal activity in Crohn’s disease. Clin. Gastroenterol. Hepatol..

[29] Omori T., Sasaki Y., Koroku M., Murasugi S., Yonezawa M., Nakamura S., Tokushige K. (2022). Serum leucine-rich alpha-2 glycoprotein in quiescent Crohn’s disease as a potential surrogate marker for small-bowel ulceration detected by capsule endoscopy. J. Clin. Med..

[30] Best W.R., Becktel J.M., Singleton J.W., Kern F. (1976). Development of a Crohn’s disease activity index. National Cooperative Crohn’s disease Study. Gastroenterology.

[31] Gralnek I.M., Defranchis R., Seidman E., Leighton J.A., Legnani P., Lewis B.S. (2008). Development of a capsule endoscopy scoring index for small bowel mucosal inflammatory change. Aliment. Pharmacol. Ther..

[32] Niv Y., Ilani S., Levi Z., Hershkowitz M., Niv E., Fireman Z., O’Donnel S., O’Morain C., Eliakim R., Scapa E. (2012). Validation of the Capsule Endoscopy Crohn’s disease Activity Index (CECDAI or Niv score): A multicenter prospective study. Endoscopy.

[33] Rosa B., Moreira M.J., Rebelo A., Cotter J. (2012). Lewis Score: A useful clinical tool for patients with suspected Crohn’s disease submitted to capsule endoscopy. J. Crohns. Colitis.

[34] Toniatti C., Arcone R., Majello B., Ganter U., Arpaia G., Ciliberto G. (1990). Regulation of the human C-reactive protein gene, a major marker of inflammation and cancer. Mol. Biol. Med..

[35] Vermeire S., Van Assche G., Rutgeerts P. (2006). Laboratory markers in IBD: Useful, magic, or unnecessary toys?. Gut.

[36] Levy I., Gralnek I.M. (2016). Complications of diagnostic colonoscopy, upper endoscopy, and enteroscopy. Best. Pract. Res. Clin. Gastroenterol..

[37] Levesque B.G., Sandborn W.J., Ruel J., Feagan B.G., Sands B.E., Colombel J.F. (2015). Converging goals of treatment of inflammatory bowel disease from clinical trials and practice. Gastroenterology.

[38] Sturm A., Maaser C., Calabrese E., Annese V., Fiorino G., Kucharzik T., Vavricka S.R., Verstockt B., van Rheenen P., Tolan D. (2019). ECCO-ESGAR Guideline for Diagnostic Assessment in IBD Part 2: IBD scores and general principles and technical aspects. J. Crohns. Colitis.

[39] Ben-Horin S., Lahat A., Amitai M.M., Klang E., Yablecovitch D., Neuman S., Levhar N., Selinger L., Rozendorn N., Turner D. (2019). Assessment of small bowel mucosal healing by video capsule endoscopy for the prediction of short-term and long-term risk of Crohn’s disease flare: A prospective cohort study. Lancet. Gastroenterol. Hepatol..

[40] Yasutomi E., Inokuchi T., Hiraoka S., Takei K., Igawa S., Yamamoto S., Ohmori M., Oka S., Yamasaki Y., Kinugasa H. (2021). Leucine-rich alpha-2 glycoprotein as a marker of mucosal healing in inflammatory bowel disease. Sci. Rep..

