# Supplementary material for: Evaluation of Crohn’s Disease Small-Bowel Mucosal Healing Using Capsule Endoscopy and Usefulness of Leucine-Rich α2-Glycoprotein
Source: Diagnostics (Basel). 2023 Feb 8;13(4):626. doi: 10.3390/diagnostics13040626 (PMC9955912; doi:10.3390/diagnostics13040626)
Supplement: Supplementary file 1 [file diagnostics-13-00626-s001.zip › diagnostics-2126901-supplementary.pdf]

Table S1. Patient Characteristics

Sex	Age (years)	Disease location	CDAI	Alb (g/dL)	CRP (mg/dL)	LRG (μg/mL)	Medication history	CE activity scores		Achievement of MH	
								LS	CECDAI	CS-MH	CE-MH
M	14	I	95	4.5	0.02	8.5	5ASA ED ADA	0	0	(-)	MH
M	42	I	70	4.4	0.11	14.2	5ASA	1036	8	(-)	Non-MH
M	37	I	38	3.8	0.33	12.4	5ASA	0	0	MH	MH
F	34	IC	327	3.6	0.11	15.2	5ASA 6MP UST	562	12	MH	Non-MH
F	39	IC	208	4.2	0.03	16.1	ED UST	233	6	MH	Non-MH
M	21	IC	155	4.2	1.33	18.5	(-)	2938	18	Non-MH	Non-MH
M	46	IC	125	3.7	0.07	19.3	5ASA ED UST	458	14	Non-MH	Non-MH
M	33	IC	107	3.6	0.20	22.2	5ASA ED ADA	1036	14	Non-MH	Non-MH
M	21	IC	100	4.6	0.1	13.8	5ASA ED	233	12	(-)	Non-MH
M	25	IC	74	4.8	0.13	9.9	5ASA ED IFX	782	13	Non-MH	Non-MH
M	20	IC	65	5.1	0.11	11.7	5ASA ED AZP IFX	3789	8	(-)	Non-MH
F	49	IC	56	4.6	0.03	9.7	5ASA AZP ADA	0	3	MH	MH
M	48	IC	43	4.5	0.14	14.4	ED AZP IFX	233	3	Non-MH	Non-MH

F	22	IC	34	4.5	0.03	8.8	5ASA AZP IFX	0	0	(-)	MH
F	33	IC	30	4.4	0.03	9.6	5ASA IFX	0	0	(-)	MH
F	25	IC	27	3.8	0.04	9.4	ED AZP IFX	0	0	(-)	MH
M	41	IC	20	5.1	0.02	11.6	5ASA ED AZP IFX	0	0	(-)	MH
M	45	IC	20	4.6	0.2	11.9	5ASA AZP	135	3	(-)	Non-MH
F	29	C	260	3.8	0.04	19.5	5ASA ED	0	0	Non-MH	MH
F	35	C	89	4.3	0.02	9.7	(-)	0	0	Non-MH	MH

I: Ileal type, IC: colonic type, C: colonic type, CDAI: Crohn's disease activity index, Alb: blood albumin level, CRP: C-reactive protein, LRG: Leucine-Rich Alpha-2 glycoprotein, 5ASA: 5-aminosalicylic acid, ED: elemental diet, 6MP: 6-mercaptopurine, AZP: azathioprine, IFX: infliximab, ADA: adalimumab, UST: ustekinumab, LS: Lewis score, CECDAI: capsule endoscopy Crohn's disease activity index, CS: ileocolonoscopy, MH: mucosal healing, CE: capsule endoscopy. "CS-MH" means the group that achieved MH within the CS observation range (terminal ileum-rectum). "CE-MH" means the group that achieved MH in both LS <135 and CECDAI <3.5.
